# Investigation of the behavior of tinnitus patients under varying listening conditions with simultaneous electroencephalography and pupillometry

**DOI:** 10.1002/brb3.3571

**Published:** 2024-06-06

**Authors:** Eser Sendesen, Didem Turkyilmaz

**Affiliations:** ^1^ Department of Audiology Hacettepe University Ankara Turkey

**Keywords:** electroencephalography, extended high frequencies, listening effort, pupillometry, tinnitus

## Abstract

**Objective:**

This study aims to control all hearing thresholds, including extended high frequencies (EHFs), presents stimuli of varying difficulty levels, and measures electroencephalography (EEG) and pupillometry responses to determine whether listening difficulty in tinnitus patients is effort or fatigue‐related.

**Methods:**

Twenty‐one chronic tinnitus patients and 26 matched healthy controls having normal pure‐tone averages with symmetrical hearing thresholds were included. Subjects were evaluated with 0.125−20 kHz pure‐tone audiometry, Montreal Cognitive Assessment Test (MoCA), Tinnitus Handicap Inventory (THI), EEG, and pupillometry.

**Results:**

Pupil dilatation and EEG alpha power during the “encoding” phase of the presented sentence in tinnitus patients were less in all listening conditions (*p* < .05). Also, there was no statistically significant relationship between EEG and pupillometry components for all listening conditions and THI or MoCA (*p* > .05).

**Conclusion:**

EEG and pupillometry results under various listening conditions indicate potential listening effort in tinnitus patients even if all frequencies, including EHFs, are controlled. Also, we suggest that pupillometry should be interpreted with caution in autonomic nervous system‐related conditions such as tinnitus.

## Graphical abstract

Topographic maps distribution of EEG alpha band in different listening conditions.

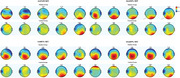

## 1 INTRODUCTION

Tinnitus is the perception of sound without an external auditory stimulus (De Ridder et al., 2021). The prevalence of tinnitus has been described in the literature, with rates ranging from 5% to 30% in various studies (McCormack et al., 2016). It can directly cause sleep disturbances (Li et al., 2022). Also, behavioral studies suggest that tinnitus has cognitive effects on individuals’ daily lives, such as problems with attention, memory, and concentration (Cho et al., 2013; Degeest et al., 2022; Hallam et al., 2004; Rossiter et al., 2006; Tegg‐Quinn et al., 2016). These cognitive problems can affect the listening skills of tinnitus patients (Kim & Phillips, 2014; Peelle, 2018; Rudner, 2016).

“Listening effort” is the management of cognitive resources used to overcome difficulties encountered while performing the listening task under difficult conditions (Francis & Love, 2020; McGarrigle et al., 2014; Peelle, 2018). Also, “listening fatigue” is defined as fatigue caused by prolonged effortful listening (Alhanbali et al., 2019; McGarrigle et al., 2014). These two situations can be grouped under the title “listening difficulty.” The listening difficulty is composed of two main headings. The first relates to stimulus‐related factors such as the resolution of the auditory stimulus (e.g., poor articulation, distorted auditory stimulus) and the listening environment (e.g., echoey, noisy) (Ohlenforst et al., 2017). The second factor is individual‐related factors such as cognitive (e.g., memory, attention) and auditory (e.g., hearing loss, auditory processing problems) skills (Peelle, 2018).

In the literature, electroencephalography (EEG) and pupillometry are widely recommended for physiologic assessment of listening effort (Fiedler et al., 2021; Haro et al., 2022; Miles et al., 2017; Sendesen et al., 2023). Pupillometry is closely related to the autonomic nervous system (ANS) response that develops as the processing load increases (Sirois & Brisson, 2014). It has been shown that pupils dilate as the signal‐to‐noise ratio (SNR) gets lower (Sirois & Brisson, 2014). It has been observed that when fatigue occurs, the increase in pupil size stops and decreases after a certain size (Hopstaken et al., 2015; Ronan McGarrigle et al., 2017; R. McGarrigle et al., 2017). EEG is assumed to be related to the central nervous system (CNS) response to increased processing load (Hall et al., 2019). An increase in activations in the alpha frequency band (8−12 Hz) has been observed during tasks requiring more active use of working memory than usual when using nontonal auditory stimuli such as syllables, words, and sentences (Alhanbali et al., 2019). In contrast, it has been shown that fatigue increases EEG alpha band activation (Hunter, 2022; Trejo et al., 2015).

There are studies on potential listening efforts in tinnitus patients (Degeest et al., 2022; Huang et al., 2022; Juul Jensen et al., 2018; Gürses et al., 2018; Sendesen & Turkyilmaz, 2024; Sendesen et al., 2023). Only a few of these studies used objective assessment methods (Juul Jensen et al., 2018; Sendesen & Turkyilmaz, 2024; Sendesen et al., 2023). Furthermore, some of the studies evaluating listening effort in tinnitus patients did not assess tinnitus patients’ extended high frequencies (EHFs) (8−20 kHz) (Degeest et al., 2022; Huang et al., 2022; Juul Jensen et al., 2018). However, EHFs have been shown to affect speech perception ability in noise (SPIN) (Motlagh Zadeh et al., 2019). Controlling for EHFs between the tinnitus and control groups might be crucial in light of the possibility that SPIN skills cause listening difficulty.

The remaining studies evaluating listening hanin tinnitus patients have not been entirely sure whether this difficulty is related to effort or fatigue (Sendesen & Turkyilmaz, 2024). Sendesen et al. (2023) addressed this in an attempt to define the listening difficulty in tinnitus patients. This study has once again confirmed the listening difficulty in tinnitus patients. However, it was emphasized that the pupil size may have changed in tinnitus patients due to ANS involvement rather than the effort needed to listen to the presented stimuli. Even when EHFs were controlled between groups, pupil size showed similar behavior (Sendesen & Turkyilmaz, 2024). To understand this behavior of pupil diameter which is important to determine the type of listening difficulty, changes in pupil size can be monitored by presenting stimuli of various difficulty levels to tinnitus patients, and the characteristics (effort or fatigue) of the listening difficulty can be checked with EEG responses simultaneously. Thus, this study aims to control all hearing thresholds, including EHFs, present stimuli of varying difficulty levels to tinnitus patients, and measure EEG and pupillometry responses to determine whether listening difficulty is effort or fatigue‐related.

## 2 MATERIALS AND METHODS

### 2.1 Participants

The tinnitus group consisted of patients who came to our university hospital complaining of tinnitus. The control group was chosen from among the volunteers who responded to our research announcement on campus. The inclusion criteria for the tinnitus group were having chronic tinnitus (more than 6 months) and having pure tone thresholds (PTTs) (between 0.125 and 8 kHz) within normal limits. The exclusion criteria for the tinnitus group were having an organic etiology of tinnitus (such as otosclerosis, endolymphatic hydrops, acoustic neurinoma, and otitis media) according to their medical history, radiologic images, and audiologic evaluation results, and not receiving any therapy or treatment for their tinnitus. For the control group, the inclusion criterion was that PTTs (between 0.125 and 8 kHz) were within normal limits. The exclusion criterion was having acute or chronic tinnitus. The common exclusion criterion for both groups was the presence of demyelinating diseases that may affect the CNS or ANS.

A total of 50 participants (23 males and 27 females) were included in the study. Three participants, one from the control group and two from the tinnitus group, were excluded from the study because their continuous EEGs were unreliable (there was a high level [≥ 80 μV] of electrical artifact in the electrodes where the continuous EEG analysis was performed). Consequently, statistical analysis was performed on 21 participants in the tinnitus group (10 males and 11 females) and 26 in the control group (12 males and 14 females).

The participants’ otoscopic and tympanometric examinations revealed normal outer and middle ear functions. A GSI‐61 audiometer and calibrated TDH‐39P (for 0.125−8 kHz) headphones, a Sennheiser HDA200 (for 9−20 kHz), and a Radioear B‐71 bone vibrator were used for pure‐tone audiometry. The behavioral pure‐tone hearing thresholds (0.125−8 kHz) were less than 15 dB HL. The Edinburgh Handedness Inventory (Veale, 2014) revealed that all participants were right‐handed. The Montreal Cognitive Assessment Test (MoCA), a screening test that evaluates general cognitive function, was administered to the participants. A score of 26 or higher indicates that the participant has a normal cognitive function (Nasreddine et al., 2003). The Tinnitus Handicap Inventory (THI) was given to the tinnitus group. All evaluations were given to each group in turn. Finally, pupillometry and EEG were performed simultaneously to assess the participants’ listening difficulty.

### 2.2 Psychoacoustic assessment of tinnitus

To prevent the patient from becoming confused by the auditory stimulus presented with tinnitus, tinnitus frequency was found using a two‐alternative forced selection procedure with stimuli presented at 30 dB SL between 0.125 and 20 kHz from the contralateral ear (Henry & Meikle, 2000). The tinnitus loudness level was then matched in 5 dB HL steps based on the participants’ ipsilateral hearing threshold at the tinnitus frequency (Henry & Meikle, 2000).

The minimum masking level (MML) was determined in 5 dB steps as the level at which the tinnitus was masked by narrowband noise, whose center frequency was the tinnitus frequency (Henry & Meikle, 2000). Participants with bilateral or central tinnitus were presented with narrowband noise on both sides of their heads. In the case of unilateral tinnitus, it was presented to the tinnitus ear.

To determine residual inhibition (RI), narrowband noise that matched the perception of center frequency tinnitus was presented bilaterally at 10 dB above the minimal masking level for 60 s (Henry & Meikle, 2000). These findings were classified as positive if the level of tinnitus perception decreased, negative if tinnitus perception did not change, or increased.

### 2.3 Tinnitus questionnaire

The THI was utilized to assess the impact of tinnitus on the participants’ daily lives (Aksoy et al., 2007; Newman et al., 1996). The THI consists of 25 questions. It assesses the subjective psychological effects of tinnitus patients. It evaluates functional, emotional, and destructive tinnitus subscales. “Yes,” “Sometimes,” and “No” are the answers. These answers are scored as “4,” “2,” and “0,” respectively, and the THI score is calculated by summing these scores from each question.

### 2.4 Speech‐in‐Noise Test

The Matrix Test (MT) (Zokoll et al., 2013) was used as a Speech‐in‐Noise Test. This test was used to determine 50% and 80% SRT levels of the participants. The MT material contains 10 words from a word group: a name, a number, an adjective, an object, and a verb (Kollmeier et al., 2015). Although there are only 50 words, many unique sentences can be created and presented to the participants by freely combining words from various word groups. Furthermore, because the sentences presented are syntactically correct but lack semantic structure, the participants’ chances of correctly guessing them are extremely low. The MT contains 30 unique lists, each containing 20 sentences. Each participant was presented with a single list to test their performance in each SNR. Since the present study tested 50% and 80% SRT (Speech Reception Threshold) levels, each participant was presented with two lists. The time required for the two lists was approximately 10 min. Sentences were initially presented using Sennheiser HDA200 headphones and an open‐ended presentation model with an SNR of 0 dB. While the white noise level in the test is constant (65 dB SPL), the speech stimulus is adaptive. During the trial, the SNR level changes proportionally depending on whether participants maintain a percentage of correct answers above or below the predetermined threshold (50% and 80% SRT levels). The presented SNR level varied according to the performance in the previously presented sentence (according to the ability to guess more or less than 50% or 80% of the words). The test result was determined by the participant's SNR level in the 20th sentence according to the predetermined SRT score (50% and 80% SRT levels).

### 2.5 Stimulus design and presentation for physiological measurements

Stimuli were created based on Miles et al.’s research (Miles et al., 2017). MT sentences were used for speech materials. The sentences in the MT (The lists associated with the sentences used during the MT previously presented to the participants were excluded.) and the multi‐talker babble noise were combined using custom MATLAB scripts. After that, the stimuli were divided into 6 and 16 logarithmic channels. The amplitude envelope was created by taking the absolute value of each channel from the Hilbert transform and dividing it by the number of channels. The extracted envelope was used to modulate noise in the same frequency band. To create noise‐vocoded sentences and background noise, each noise band was recombined. After generating noise‐vocoded speech in this way, the root means square levels of the sentences and background noise were equalized using MATLAB. Consequently, the duration of the stimulus was 6 s. It included noise from 0 to 1 s, noise‐vocoded speech from 1 to 4.5 s, and noise from 4.5 to 6 s.

EEG and pupil diameters were recorded simultaneously in a sound‐isolated room during the speech recognition task. Noise‐vocoded speech with 6 and 16 channels was generated at 50% and 80% SRT levels of the stimuli determined by the MT. These were labeled “6ch50% SRT,” “16ch50% SRT,” “6ch80% SRT,” and “16ch80% SRT.” Each stimulus had a duration of 9 min. Two‐minute breaks were given after each stimulus. Thus, the time allocated for each participant was approximately 45 min. To avoid the order effect, each stimulus was presented to each participant differently. Participants were instructed to repeat the sentence when the noise stopped. The participants’ active attendance to the experiment (repeating the sentences) was confirmed by the microphone and camera placed in the room.

### 2.6 EEG recording and postprocessing

Continuous EEG data were recorded using a NuAmps II Neuroscan amplifier with 21 channels. Nineteen electrodes were placed on the scalp using the standard 10−20 configuration. Both earlobes’ electrical activity was recorded. A2 was designated as the reference electrode. All electrode impedances were kept below 5 kiloohms during recording. Continuous EEG data were filtered with a notch filter to remove 50 Hz artifacts.

MATLAB 2016a was used to perform postprocessing on EEGlab. Eye blinks were automatically identified and removed with EEGlab. The alpha band was revealed by filtering continuous EEG data between 8 and 12 Hz (Miles et al., 2017). The envelope of each EEG segment was transformed using the Hilbert transform. During the encoding period (Miles et al., 2017) (1 s duration ending 200 ms before the end of the sentence) and baseline in noise (300−800 ms after the noise onset), the absolute value of the alpha band was extracted from the parietal electrodes (P3, P4, and Pz) trial by trial. Worsening speech or adding background noise appears to modulate parietal alpha power, suggesting that it may be a neural correlate of listening effort (Decruy et al., 2020; McMahon et al., 2016). Therefore, parietal electrodes were used for EEG analysis. The relative percent change for each trial was calculated by subtracting mean alpha power during encoding from mean alpha power during baseline and dividing by mean alpha power during baseline (Miles et al., 2017). The percentage change from the baseline was calculated by multiplying the result by 100.

### 2.7 Pupil recording and postprocessing

The EyeLink 1000 Plus system (Research, 2010) was used to sample the right pupil at 1000 Hz. All stimuli used in the study were presented using Experiment Builder software. Pupillometry was performed at the light levels in the room where the device was initially calibrated (31.5 asb, 1 asb = 0.31831 cd/m^2^). Before each experiment, the device was calibrated using the 9‐point calibration grid on the screen. The pupil sizes are measured in pixels by the eye tracker system. The sampling frequency was 1000 hertz (i.e., every 1 ms). The laboratory had soft lighting and was soundproofed. The participants sat in front of a flat 22‐inch LED monitor with a 1920 × 1080 pixel resolution and a refresh rate of 60 Hz. Their heads were stabilized with a chin rest to improve calibration accuracy and keep the eye‐to‐monitor distance constant at 60 cm. The first five stimuli were excluded for each listening condition from the data analysis to avoid potential effects such as habituation, excitement, and arousal. Participants were instructed to focus on the fixation cross (Miles et al., 2017). At the same time, they were instructed to listen to the sentences and avoid blinking during the sentence presentation.

Using the R package PupilPre, the preprocessing methods were applied to the raw pupil data. First, the raw pupil data were deblinked (blink removal process) from 100 ms before the start of a blink to 100 ms after the end of a blink. To reduce the number of lost samples and blink artifacts in the remaining accepted trials, linear interpolation was used. We calculated each trial's baseline pupil size by averaging the pupil size from 500 ms before the stimuli to their onset. Then, we performed standard baseline subtraction for each trial to compare the relative changes in pupil size. We downsampled the data to 50 Hz at the end of the preprocessing. Only the right eye's data were preprocessed and analyzed. Previous pupillometry studies have referenced the right eye in right‐handed dominant participants (Fotiou et al., 2000). Each participant's trials were averaged for each condition. The noise (0−1 s) and encoding period (2−6 s) were critical points for analysis (Miles et al., 2017). The mean pupil size during the noise phase (0−1 s) was subtracted from the maximum pupil dilatation (MPD) during the encoding phase for each trial's relative percent change. This result is then divided by the baseline value (mean pupil size during the noise phase [0−1 s]). The percentage change from the baseline point is then calculated by multiplying this value by 100. Thus, the MPD relative to the baseline was calculated.

### 2.8 Statistical methods

The sample size for the study was determined using the G*Power program (Kang, 2021). Based on the mean and standard deviation values from the groups in the pilot study, this study should include 17 participants (effect size = 1.16) from each group, with a 5% type I error level and 95% power to detect a minimal, statistically significant difference (*p* < .05). The data were evaluated using the SPSS version 26 package program (IBM Inc.). The independent sample *t*‐test was used to compare MPD and EEG alpha power between groups. Levene's test was used to verify the assumption of variance homogeneity. Pearson's correlation coefficient was also used to examine the relationship between MPD, EEG alpha band, and MoCA scores in all participants, as well as the relationship between these scores and THI scores in the tinnitus group only.

## 3 RESULTS

### 3.1 Descriptive statistics

**FIGURE 1:**
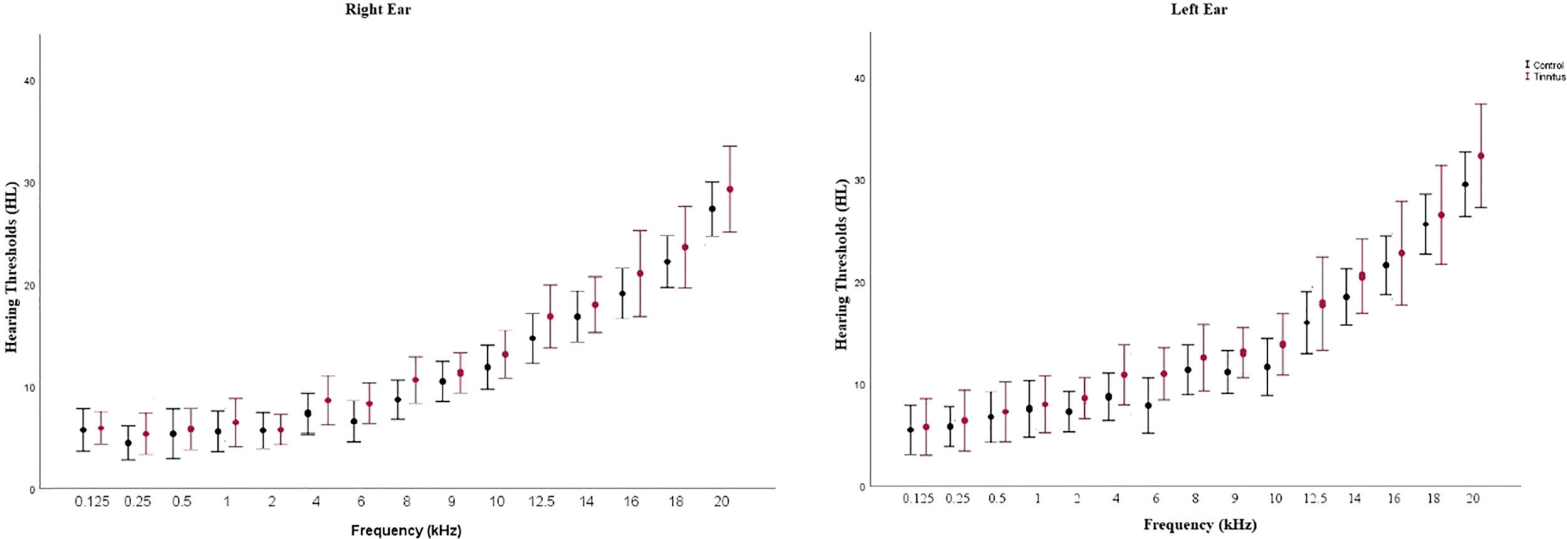
The average hearing thresholds for each group's right and left ears.

The age distribution of the groups reveals that the tinnitus group had an average age of 24.89 ± 3.17 (23−33) years. The control group's mean age was 23.57 ± 2.94 (22−29) years. Age and sex differences between groups were not significant (*p* > .05). All PTTs were not significantly different between groups (*p* > .05) for each frequency range (0.125−20 kHz). There were 18 patients with bilateral tinnitus and 3 with unilateral (two in the right ear and one in the left ear). Mean hearing thresholds for each frequency are shown in Figure 1.

The tinnitus patients’ average THI score was 40.6 ± 9.9. The tinnitus group had a mean MoCA score of 27.47 ± 1.07 (26−30), while controls had 28.04 ± 0.97 (26−30). The independent sample *t*‐test revealed no difference in MoCA scores between groups (*p* > .05). Table 1 shows the participants’ tinnitus characteristics. Table 2 shows the mean SRT scores statistical evaluation for the groups.

**TABLE 1:** The tinnitus group's (*n* = 21) characteristics and MoCA scores.

Subject no	Tinnitus location	Tinnitus pitch	TLL	MML	RI	THI score
1	Bilateral	8 kHz	35 dB	25 dB	+	42
2	Bilateral	8 kHz	25 dB	20 dB	+	44
3	In the head	8 kHz	45 dB	30 dB	+	28
4	Right ear	9 kHz	35 dB	15 dB	+	38
5	In the head	8 kHz	55 dB	40 dB	+	42
6	Bilateral	9 kHz	40 dB	35 dB	–	34
7	Bilateral	6 kHz	25 dB	25 dB	+	46
8	In the head	8 kHz	35 dB	30 dB	+	36
9	Bilateral	9 kHz	20 dB	15 dB	+	28
10	Right ear	4 kHz	45 dB	35 dB	+	40
11	Bilateral	8 kHz	65 dB	45 dB	+	52
12	Bilateral	6 kHz	30 dB	25 dB	+	44
13	Left ear	5 kHz	15 dB	15 dB	–	62
14	Bilateral	7 kHz	25 dB	20 dB	+	46
15	Left ear	4 kHz	55 dB	45 dB	+	32
16	Bilateral	9 kHz	15 dB	10 dB	+	26
17	Bilateral	8 kHz	25 dB	20 dB	+	34
18	In the head	6 kHz	45 dB	45 dB	–	42
19	Bilateral	4 kHz	55 dB	40 dB	+	56
20	Bilateral	9 kHz	35 dB	30 dB	+	28
21	In the head	8 kHz	25 dB	25 dB	+	54

Abbreviations: MML, minimum masking level; RI, residual inhibition; THI, Tinnitus Handicap Inventory; TLL, tinnitus loudness level.

**TABLE 2:** Mean SRT scores and statistical evaluation resulting from the matrix test for tinnitus (*n* = 21) and control groups (*n* = 26).

	Tinnitus group	Control group	
Variable	Mean ± Std. (dB SNR)	Mean ± Std. (dB SNR)	*p*‐value
**50% SRT**	1.71 ± 0.87	1.63 ± 0.71	>.05
**80% SRT**	2.41 ± 0.82	2.37 ± 0.64	>.05

Abbreviations: SRT, speech reception thresholds; Std., standard deviation.

### 3.2 EEG alpha power differences between groups

**FIGURE 2:**
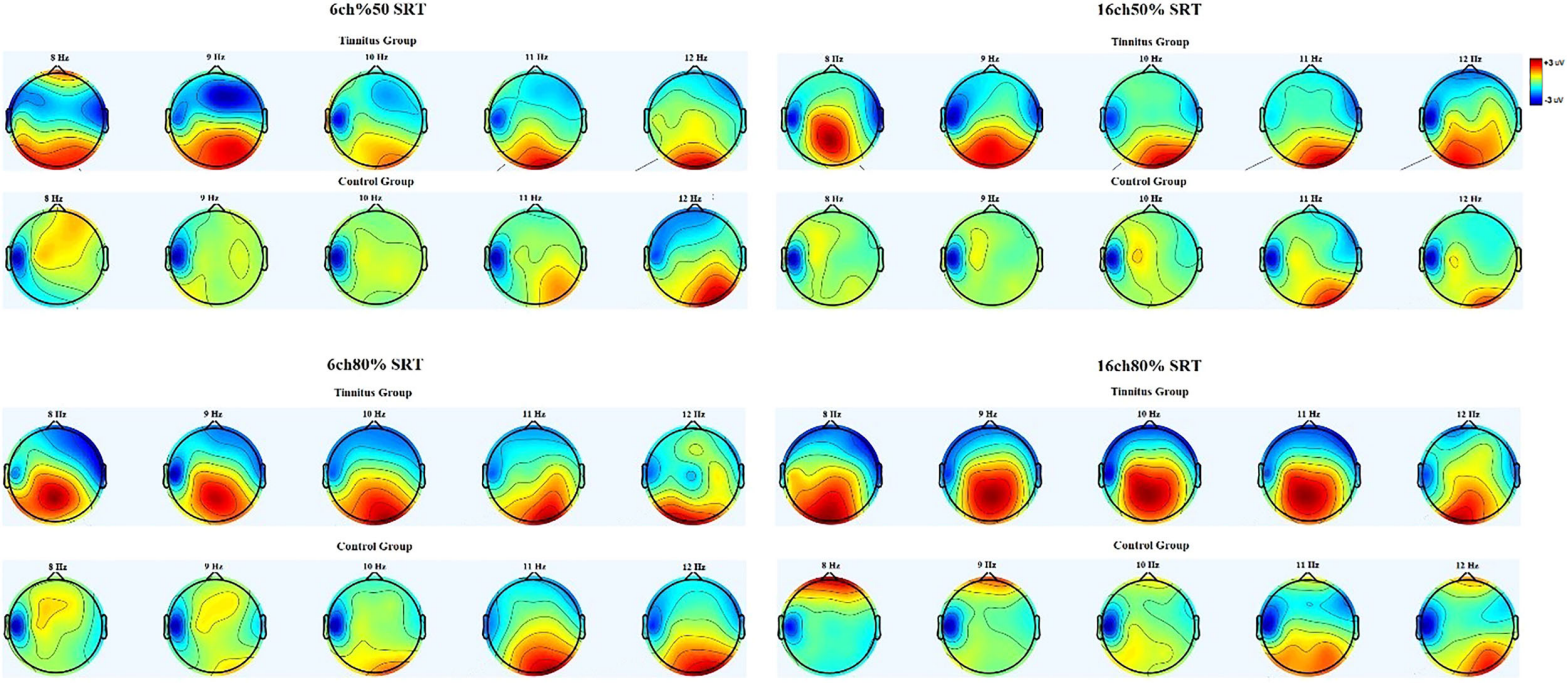
Topographical maps of EEG alpha band response for each listening condition in tinnitus (*n* = 21) and control groups (*n* = 26).

Table 3 shows the mean EEG alpha power change relative to baseline and a statistical evaluation of the groups for each SRT listening condition. The independent sample *t*‐test revealed that for all listening conditions, the increase in the EEG alpha band was significantly lower in the tinnitus group (*p* < .01). Figure 2 displays topographical maps of EEG alpha band response for each listening condition in the groups.

**TABLE 3:** Statistical analysis of differences in mean EEG alpha power change for each listening condition between tinnitus (*n* = 21) and control (*n* = 26) groups.

	Tinnitus group	Control group	
Listening conditions	Mean ± Std. (%)	Mean ± Std. (%)	*p*‐value
**6ch50% SRT**	113.92 ± 84.26	183.60 ± 61.01	.01*
**16ch50% SRT**	116.14 ± 63.35	200.86 ± 95.31	.006*
**6ch80% SRT**	88.50 ± 41.54	174.66 ± 78.38	.007*
**16ch80% SRT**	124.23 ± 67.70	210.69 ± 96.71	.007*18

Abbreviation: Std., standard deviation.

*Independent samples *t*‐test. Bold *p* values: statistically significant difference.

In addition, the effect of the change in EEG alpha power on MoCA scores under varying listening conditions for all participants and on THI for the tinnitus group was evaluated. Pearson's correlation coefficient found no statistically significant correlation between EEG alpha power change in any listening condition and THI scores, nor between MoCA (*p* > .05).

### 3.3 MPD differences between groups

Table 4 shows the mean MPD relative to the baseline for all listening conditions and a statistical evaluation of the groups. The independent sample *t*‐test revealed that MPD was significantly lower in the tinnitus group (*p* < .05).

**TABLE 4:** Statistical analysis of differences in MPD for each listening condition between tinnitus (*n* = 21) and control (*n* = 26) groups.

	Tinnitus group	Control group	
Listening conditions	Mean ± Std. (%)	Mean ± Std. (%)	*p*‐value
**6ch50% SRT**	9.1 ± 0.3	10.7 ± 0.3	**<.001**
**16ch50% SRT**	8.7 ± 0.3	10.2 ± 0.3	**<.001**
**6ch80% SRT**	9.6 ± 0.3	10.5 ± 0.4	**.03**
**16ch80% SRT**	8.4 ± 0.3	10.3 ± 0.2	**<.001**

Abbreviation: Std., standard deviation.

Bold *p* values: statistically significant difference.

The effect of MPD on MoCA scores under varying listening conditions for all participants and on THI for the tinnitus group was evaluated. Pearson's correlation coefficient showed no statistically significant correlation between MPD in any listening condition and THI scores or MoCA (*p* > .05).

## 4 DISCUSSION

The main aim of this study was to evaluate EEG and pupillometry responses in tinnitus patients by presenting stimuli of varying difficulty levels to determine whether listening difficulty is related to effort or fatigue. According to the findings, tinnitus patients had a lower increase in EEG alpha band power and MPD during the encoding phase of the speech stimulus. To the best of our knowledge, this is the first study to investigate listening difficulty in tinnitus patients using EEG and pupillometry with stimuli of varying difficulty levels by carefully matching the auditory sensitivity of participants across the two groups in the 0.125−20 kHz range to control the potential effect of hearing loss on listening skills.

In previous studies, it has been reported that EEG alpha band power is highest when a slightly acoustically distorted speech stimulus is presented and lowest when a severely acoustically distorted stimulus is presented (Fiedler et al., 2021; Miles et al., 2017). In studies conducted with speech stimuli of various difficulty levels, EEG alpha band power increased once the stimulus's difficulty level reached a certain point. When the stimulus's difficulty level reached more severe, the EEG alpha band power started to decrease (Hunter, 2022; Li et al., 2020; Obleser & Weisz, 2012; Obleser et al., 2012). As the difficulty of the speech stimulus increases, the acoustic cues required for the participants’ comprehension of this stimulus will begin to disappear. After that, the number of neural resources in the brain allocated to analyzing the speech stimulus will decrease (Hunter, 2022; Obleser & Weisz, 2012). This condition is called fatigue. It has been demonstrated that as a result of fatigue, the general neural inhibition mechanism in the brain increases, and the size of neural resources allocated for speech stimulus encoding in the brain decreases, increasing EEG alpha band power (Eoh et al., 2005; Li et al., 2020).

On the other hand, the ANS has two components: the sympathetic nervous system (SNS) and the parasympathetic nervous system (PNS). It has been proposed that pupil diameter increases with increasing SNS activity, whereas pupil diameter decreases with increasing PNS activity (Pfeifer et al., 1983; Turnbull et al., 2017). When presented with an acoustically distorted speech stimulus, pupil diameter increases with increased SNS activity. However, it has been demonstrated that when the difficulty level of the speech stimulus increases to the point where the participant is unable to provide the necessary auditory cues for the presented stimulus, the increase in pupil diameter stops, and the PNS leads to a decrease in pupil size due to fatigue (Sendesen et al., 2023; Pfeifer et al., 1983; Turnbull et al., 2017).

Juul Jensen et al. (2018) were unable to determine whether the difference between the groups was due to effort or fatigue in their pupillometry study. Sendesen and Turkyilmaz (2024) also used normal hearing thresholds, including EHF, between the groups in their pupillometry study, but were unable to determine whether the listening difficulty in tinnitus patients was related to fatigue or effort. In the present study, the tinnitus group's alpha band power increase was lower in all listening conditions. This finding supports Degeest et al. (2017) (using dual‐task paradigm) and Sendesen et al. (2023) (using EEG and pupillometry) hypothesis's of a potential listening effort in tinnitus patients. If this difference was due to fatigue, the brain's general inhibition mechanism would dominate, and the EEG alpha band power would increase since more neural resources would not be needed (Hunter, 2022). Furthermore, the tinnitus group's EEG alpha power was low in all listening conditions. In other words, even if the listening condition became difficult due to the acoustic distortion of the speech stimulus and the prolonged duration of the experiment, the tinnitus patients continued to listen, and no fatigue was observed.

According to the topographic maps, the tinnitus group's EEG alpha power decreased during the encoding phase of the speech stimulus, and this decrease was more spread across the electrodes than in the control group. Previous studies demonstrated that increased activity in specific brain areas reduces EEG alpha power (Klimesch et al., 2007). The decrease in EEG alpha band power in the frontocentral and frontoparietal electrodes in the tinnitus group compared to the control group may indicate that the tinnitus group has more activity in these regions. These regions are directly related to processes such as attention, complex problem‐solving, and working memory (Fink et al., 2009). Higher activity levels in this region in tinnitus patients may indicate greater effort.

In pupillometry, MPD was lower in the tinnitus group in all listening conditions. At first glance, this result could be interpreted as tinnitus patients failing to provide sufficient auditory cues to encode the incoming speech stimulus and giving up encoding the stimulus due to fatigue (Sendesen & Turkyilmaz,2024 ;Sendesen et al., 2023). In the case of listening effort, pupil diameter has been shown to increase in healthy subjects with PNS suppression and SNS activation (Sirois & Brisson, 2014). However, neurophysiological theory explains that tinnitus alters normal ANS behavior (Jastreboff, 2004). According to this theory, when tinnitus is bothersome, the SNS is more active in tinnitus patients than in healthy people. During the experiment, the stimulus presented to the participants was at least 65 dB SPL. MML values for all tinnitus participants were less than 65 dB SPL. So, the participants were unable to perceive their tinnitus during the experiment. This may have resulted in a lower stress level for tinnitus during auditory stimulus presentation and higher PNS activation compared to the control group. As a result, increased PNS activity may have decreased pupillary diameter, overshadowing the listening effort that may occur in tinnitus patients and causing pupillometry results to be misinterpreted as fatigue. In addition to the recent study (Sendesen et al., 2023), which evaluated listening skills in tinnitus patients simultaneously with EEG and pupillometry, we provided different listening conditions in the present study. This allowed us to see the pupil's response to easy to difficult listening conditions. If the results were related to fatigue, the PNS would become dominant as the listening condition became more difficult, and a decrease in pupil diameter would be expected due to fatigue. However, decreased MPD was seen in both the tinnitus and control groups in the easiest listening condition, 16ch80% SRT, compared to the most difficult listening condition, 6ch50% SRT, in accordance with the effort. As a result, this can be regarded as evidence that pupillometry results in the tinnitus group supported the listening effort. Also, pupillometry may not provide reliable results in ANS‐related conditions such as tinnitus, and the findings should be interpreted with caution.

There are essentially two primary categories of factors that influence listening effort (Alhanbali et al., 2019; McGarrigle et al., 2014). These can be categorized as stimulus‐related and individual‐related. Because stimulus‐related factors in listening effort were also valid (the same stimulus was used in the tinnitus control group) in the control group, these factors are not considered to be a possible cause of listening effort in tinnitus patients. Previous studies have shown that the reason for listening effort in tinnitus patients is that their attention is on the tinnitus rather than the speech stimulus (Degeest et al., 2017; Degeest et al., 2022). However, considering that the level of the speech stimulus presented is at least 65 dB SPL and that tinnitus patients’ MML levels are lower than 65 dB SPL, it is highly likely that the participants cannot perceive their tinnitus. Furthermore, it is assumed that if participants’ attention is directed to the tinnitus rather than the speech stimulus during the EEG assessment, the number of neural resources allocated to encoding the presented speech stimulus will decrease. As a result, cortical inhibition is expected to increase and EEG alpha power to increase during stimulus encoding in the tinnitus group (Klimesch et al., 2007).

It has been demonstrated that tinnitus causes anxiety in individuals and that anxiety influences the use of cognitive resources (Bhatt et al., 2017). The current study used the THI to provide baseline information about participants’ anxiety related to tinnitus. However, no correlation was found between THI scores and EEG alpha band power or pupil diameter increase percentages in any listening condition. According to this finding, there seems to be no relationship between tinnitus‐related anxiety and listening effort. However, since this conclusion cannot be supported by a questionnaire such as the THI, which provides only basic information about anxiety related to tinnitus, the results should be interpreted cautiously.

Previous research has found that tinnitus patients have weaker working memory and general memory processes, potential factors for listening effort (Rudner, 2016). Therefore, we assessed the relationship between pupillometry, EEG data, and MoCA scores, which are closely associated with general memory processes and working memory. No correlation was found between MoCA scores, pupil diameter, and EEG alpha band power in either the tinnitus group or the control group. Also, a previous study, which supports our findings, failed to link listening effort to working memory and general memory processes in tinnitus patients (Degeest et al., 2022). Although MoCA is an assessment method closely related to cognitive skills, it is thought that using MoCA results alone to make a definitive judgment about cognitive skills is difficult. Future studies may investigate the relationship between cognitive skills and listening effort using more comprehensive test methods.

Hearing loss has also been shown to be one of the individual‐related factors that contribute to listening effort (McGarrigle et al., 2014; Peelle, 2018). To eliminate this potential confounding variable, the hearing thresholds of the participants were matched across the entire frequency range. Although hearing thresholds did not differ between groups, studies have found differences in the central auditory pathways in tinnitus patients (Sendesen et al., 2021; Sendesen et al., 2022; Colak et al., 2024). Differences in the central auditory system have been shown to influence listening effort in children (Danneels et al., 2021). As a result, the difference in central auditory processing skills may be one of the reasons for the listening effort in tinnitus patients.

The limitations of the study can be listed as follows: Previous research has demonstrated that listening effort and vocabulary size are closely related (Zekveld et al., 2011). Although age, one of the variables that gives an idea about vocabulary, does not differ between the groups, it is not possible to argue that there is no difference between the participants without using a method that directly evaluates vocabulary. Furthermore, while we tried to control for differences in the peripheral hearing system between groups, future research may consider the possibility that suprathreshold differences, such as hidden hearing loss, may also affect listening effort. It is also known that RI in tinnitus patients is closely related to inhibition mechanisms in the CNS (Galazyuk et al., 2019). Since the number of participants with negative RI was not enough (three participants) in the present study, we could not make a statistical evaluation of the relationship between RI and listening effort. Future studies may design their methods to evaluate the effect of RI on listening efforts. In our study, the EEG alpha band was calculated in the range of 8−12 Hz. However, previous studies suggest that 8−10 Hz of the alpha band may provide more detailed information about attention processes, and 10−12 Hz may provide more detailed information about language skills such as semantic processing (Klimesch, 1999, 2012). In future studies, the EEG alpha band may be evaluated in different frequency ranges to obtain more insight into the pathophysiology of listening effort in tinnitus patients.

In all listening conditions, the increase in EEG alpha band power during the encoding phase of the speech stimulus was lower in tinnitus patients. In accordance with our hypothesis, these findings were interpreted in favor of a potential listening effort in tinnitus patients. On the other hand, the increase in MPD during the encoding phase of the speech stimulus was lower in tinnitus patients in all listening conditions, which at first glance could be interpreted as fatigue rather than effort, but this may be due to the predominance of PNS in tinnitus during the experiment. Furthermore, the findings were interpreted in favor of listening effort in tinnitus patients, as the MPD changes based on the difficulty level of the listening conditions were consistent with listening effort. According to our findings, EEG and pupillometry results under various listening conditions indicate potential listening effort in tinnitus patients even if all frequencies, including EHFs, are controlled. Also, pupillometry results should be carefully interpreted in conditions like tinnitus related to the ANS.

## AUTHOR CONTRIBUTIONS

Eser Sendesen: Conceptualization; investigation; writing—original draft; methodology; writing—review and editing; formal analysis; data curation; resources. Didem Turkyilmaz: Conceptualization; writing—review and editing; methodology; project administration; supervision.

## FUNDING INFORMARTION

This research did not receive any specific grant from funding agencies in the public, commercial, or not‐for‐profit sectors.

## CONFLICT OF INTEREST STATEMENT

The authors declare that they have no conflict of interest.

### ETHICAL APPROVAL

Ethical approval for this study was obtained from Non‐Interventional Clinical Research Ethics Committee (GO23/286) and completed in conformity with the standards set by the Declaration of Helsinki.

### INFORMED CONSENT

Informed consent was obtained from all individual participants included in the study.

### PEER REVIEW

The peer review history for this article is available at https://publons.com/publon/10.1002/brb3.3571

## Data Availability

The data that support the findings of this study are available from the corresponding author (E.S.), upon reasonable request.
